# Technical development and feasibility of a reusable vest to integrate cardiovascular magnetic resonance with electrocardiographic imaging

**DOI:** 10.1186/s12968-023-00980-7

**Published:** 2023-12-04

**Authors:** Matthew Webber, George Joy, Jonathan Bennett, Fiona Chan, Debbie Falconer, Hunain Shiwani, Rhodri H. Davies, Gunther Krausz, Slobodan Tanackovic, Christoph Guger, Pablo Gonzalez, Emma Martin, Andrew Wong, Alicja Rapala, Kenan Direk, Peter Kellman, Iain Pierce, Yoram Rudy, Ramya Vijayakumar, Nishi Chaturvedi, Alun D. Hughes, James C. Moon, Pier D. Lambiase, Xuyuan Tao, Vladan Koncar, Michele Orini, Gabriella Captur

**Affiliations:** 1 Barts Heart Centre, Barts Health NHS Trust, West Smithfield, London, ECIA 7BE UK; 2 Institute of Cardiovascular Science, University College London, Huntley Street, London, WC1E 6DD UK; 3 Centre for Inherited Heart Muscle Conditions, Department of Cardiology, Royal Free London NHS Foundation Trust, Pond Street, London, NW3 2QG UK; 4 Medical Research Council Unit for Lifelong Health and Ageing at UCL, University College London, 1-19 Torrington Place, London, WC1E 7HB UK; 5 g.Tec Medical Engineering GmbH, Siernigtrabe 14, 4521 Schiedlberg, Austria; 6 ELEM Biotech, S.L, Barcelona, Spain; 7 Department of Computer Applications in Science and Engineering, Barcelona Supercomputing Center (BSC), 08034 Barcelona, Spain; 8 Department of Information and Communication Technologies, Physense, Universitat Pempeu Fabra, Barcrlona, Spain; 9 National Heart, Lung, and Blood Institute, National Institutes of Health, Bethesda, MD 20892 USA; 10 Cardiac Bioelectricity and Arrhythmia Center, Washington University, St. Louis, MO 63130 USA; 11 Department of Biomedical Engineering, Washington University, St. Louis, MO 63130 USA; 12 École Nationale Supérieure des Arts et Industries Textiles, 2 allée Louise et Victor Champier, 59056 Roubaix CEDEX 1, France

**Keywords:** Cardiovascular magnetic resonance imaging, Electrocardiographic imaging, Electrophysiology, Feasibility, Reproducibility

## Abstract

**Background:**

Electrocardiographic imaging (ECGI) generates electrophysiological (EP) biomarkers while cardiovascular magnetic resonance (CMR) imaging provides data about myocardial structure, function and tissue substrate. Combining this information in one examination is desirable but requires an affordable, reusable, and high-throughput solution. We therefore developed the CMR-ECGI vest and carried out this technical development study to assess its feasibility and repeatability in vivo.

**Methods:**

CMR was prospectively performed at 3T on participants after collecting surface potentials using the locally designed and fabricated 256-lead ECGI vest. Epicardial maps were reconstructed to generate local EP parameters such as activation time (AT), repolarization time (RT) and activation recovery intervals (ARI). 20 intra- and inter-observer and 8 scan re-scan repeatability tests.

**Results:**

77 participants were recruited: 27 young healthy volunteers (HV, 38.9 ± 8.5 years, 35% male) and 50 older persons (77.0 ± 0.1 years, 52% male). CMR-ECGI was achieved in all participants using the same reusable, washable vest without complications. Intra- and inter-observer variability was low (correlation coefficients [*r*_s_] across unipolar electrograms = 0.99 and 0.98 respectively) and scan re-scan repeatability was high (*r*_s_ between 0.81 and 0.93). Compared to young HV, older persons had significantly longer RT (296.8 vs 289.3 ms, *p* = 0.002), ARI (249.8 vs 235.1 ms, *p* = 0.002) and local gradients of AT, RT and ARI (0.40 vs 0.34 ms/mm, *p* = 0,01; 0.92 vs 0.77 ms/mm, *p* = 0.03; and 1.12 vs 0.92 ms/mm, *p* = 0.01 respectively).

**Conclusion:**

Our high-throughput CMR-ECGI solution is feasible and shows good reproducibility in younger and older participants. This new technology is now scalable for high throughput research to provide novel insights into arrhythmogenesis and potentially pave the way for more personalised risk stratification.

*Clinical trial registration:* Title: Multimorbidity Life-Course Approach to Myocardial Health—A Cardiac Sub-Study of the MRC National Survey of Health and Development (NSHD) (MyoFit46). National Clinical Trials (NCT) number: NCT05455125. URL: https://clinicaltrials.gov/ct2/show/NCT05455125?term=MyoFit&draw=2&rank=1

**Supplementary Information:**

The online version contains supplementary material available at 10.1186/s12968-023-00980-7.

## Background

Across the world, the annual burden of sudden cardiac death (SCD) is between 4–5 million cases/year [1] and malignant ventricular arrythmias account for around 80% of this [2, 3]. Despite the availability of implantable cardioverter defibrillators (ICD), 15–20% of all deaths in Western society continue to be attributed to SCD [4]. The majority of these victims will have subtle or more overt cardiac structural abnormalities by cardiovascular magnetic resonance (CMR) imaging [5]. ICDs pose specific morbidity and mortality risks once fitted and the challenge therefore is in identifying whether the specific cardiac structural abnormality identified will put an individual at higher risk of SCD or not [6]. Detailed electrophysiological (EP) mapping procedures can be used to quantify this risk but EP studies are time consuming, invasive and limited to specialist centres [7]. Non-invasive EP studies are possible via the process of electrocardiographic imaging (ECGI), but to date we are still missing a radiation-free, cost-effective, and high-throughput solution.

ECGI is the process of combining cardiac and torso geometry with multiple body surface potentials to generate epicardial electrograms and panoramic maps of cardiac excitation [8–10]. Because ECGI is high resolution and corrected for anatomy, ECGI permits the detection of previously unobtainable and information-rich electrical phenomena otherwise missed by the conventional 12-lead ECG [7]. It has been extensively validated in ex vivo animal studies using a torso-tank experimental method [11, 12] and in several animal experiments *in vivo* [13, 14]. It has been shown to accurately correlate simultaneously with invasive EP mapping data [15] as well as in the identification of the origin of ventricular tachycardia (VT) [16, 17] and areas of low voltage typically associated with fibrosis [18].

Existing commercial ECGI solutions for healthcare are only viable with radiation-positive CT where they are single-use and costly (list price > $2000 per vest [15]). CMR combines data on cardiac structure and function with myocardial tissue characterisation [19]. This imaging modality therefore provides us with the only radiation free, non-invasive technique that can be applied to generate epicardial maps through ECGI in combination with multi-parametric cardiac data. ECGI technology has been applied to CMR research in patients with amyloidosis [20] and arrhythmogenic cardiomyopathy [21] using a Biosemi ECGI system composed of 24 adhesive electrode strips. However, this method is not reusable, and setup can be time-consuming. Therefore, none of the currently available CMR integrated ECGI solutions are optimized for high-throughput, cost-effective CMR research, let alone for roll-out to healthcare.

We identified this unmet clinical need and therefore sought to develop for the CMR community a CMR-safe, wearable, washable and re-usable ECGI vest that would be easy to don and doff with minimal patient discomfort. In this technical development and feasibility study, we present our initial results for this first-in-human use obtained with our CMR-ECGI vest. We sought to assess its performance in 4 ways: (a) feasibility of the workflow and time to deploy; (b) repeatability; (c) comparing conventional 12-lead ECG findings to ECGI parameters; (d) Assessing its ability to detect predicted age-related differences in electrical activation/repolarization.

## Methods

### Study design and population

Older age participants were recruited from MyoFit46–the cardiovascular sub-study of the National Survey of Health and Development (NSHD) (Research Ethics Committee [REC] number: 19/LO/1744, National Clinical Trial [NCT] number: NCT05455125). Participant recruitment, study protocol, data collection, storage and analysis for MyoFit46 has been described previously [22]. Young healthy volunteers were recruited (though advertising channels to staff and students at University College London) as part of an ongoing clinical research study (REC number: 21/NW/0333, NCT number: NCT05026112). Exclusion criteria were claustrophobia, presence of cardiac implantable electronic devices or atrial fibrillation.

### Vest technology and development

#### Electrodes

Conventional ECG electrodes are made out of silver-silver chloride (Ag/AgCl) and they conduct surface ECG signals through the metallic conductor with a layer of electrolyte gel usually separating the conductor from the skin [23–25]. These are easily damaged, not suitable for re-use and can cause skin irritation [26]. Dry electrode textile-based sensors utilising conductive polymers are superior to their metallic counterparts in that they are comfortable, stretchable, gel-free and can be washed and therefore re-used multiple times [27–29]. Textile-based electrodes were therefore the optimum solution for our CMR-ECGI solution. Bespoke electrodes were fabricated at the École Nationale Supérieure des Arts et Industries (ENSAIT), Roubaix, France. They are made from silver-coated polyamide yarn (Madeira HC40, Madeira Garnfabrik GmbH, Freiburg, Germany) with linear resistance less than 100 Ω/m embroidered onto a textile substrate [30]. These ‘dry’ electrodes have been previously tested for electrical characterization, ECG signal quality, surface resistivity (kΩ) and washability [28, 31, 32].

#### Textile garment

The ECGI garment is made from 100% cotton which was chosen due to its comfort, breathability, its ability to withstand high temperatures during wash cycles and its durability to the natural wear and tear that is expected from high-throughput clinical research use. In order to obtain an optimal ECG signal from the textile embroidered electrodes, the conductive yarn-based electrodes had previously been tested on various alternative textile substrates during prototyping [27, 30, 33–35].

#### Vest design

**Fig. 1:**
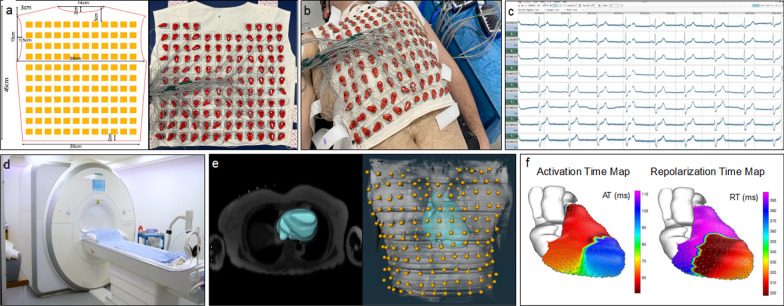
Design specifications of the CMR-ECGI vest and bird’s eye view of the high-throughput CMR-ECGI workflow. **a** The washable and re-usable CMR-ECGI vest is a cotton garment embroidered with 256 dry square textile electrodes (2 × 2 cm) and is designed to fit to a wide range of body habitus and size. Each embroidered electrode has graphite-snap connectors at the centre and Velcro fasteners at the side. **b** Fully mounted anterior electrode vest with 127 anterior ECG leads attached. The CMR-ECGI vest is fitted to the anterior and posterior torso of the participant in the supine position to allow for a 5-min recording at rest. **c** Multiple body surface potentials are collected and amplified using a multi-channel biosignal amplifier. **d** The participant proceeds to comprehensive CMR imaging at 3T. **e** The trans-axial anatomical HASTE stack allows for cardiac segmentation and electrode landmark localisation to create a detailed model of heart and torso geometry. **f** Data from the body surface potentials are then combined with the heart and torso geometry to solve the inverse solution of ECGI generating epicardial electrograms and isochrone maps of cardiac activation and repolarization. *AT* activation time, *RT* repolarization time, *CMR* cardiovascular magnetic resonance, *ECGI* electrocardiographic imaging, *HASTE* Half-Fourier acquisition single-shot turbo spin echo, *T* Tesla

Our CMR-ECGI solution is comprised of an electrode and mirror vest both made out of cotton and consists of identical front and back portions. Design specifications of the vest can be found in Fig. 1**.**

The electrode vest is embroidered with 256 silver-coated polyamide yarn-based dry square electrodes of size 2 × 2 cm. A graphite-snap connector is centrally embedded into each electrode allowing for lead connection. The electrode vest front and back portions are held together by Velcro fasteners. The electrode vest (minus the ECG leads) is fully washable. Our testing showed it retains ECG signal quality after > 50 wash cycles in a domestic laundry machine or > 200 Clinelle ™ wipe cycles, making each vest reusable in > 250 patients (further tests ongoing to date). The electrode vest components (minus the ECG leads) are also fully CMR compatible and we have tested individual components as well as the fully embroidered garment inside the CMR environment up to 3 Tesla (T) first using the T1 Mapping and extracellular volume (ECV) standardisation phantom (T1MES) [36], followed by healthy volunteer tests in vivo, to exclude heating and any image artefacts. However, to avoid the need to connect and disconnect the 256 ECG leads after each recording thus permitting rapid donning/doffing of the vest in a healthcare or research setting, our CMR-ECGI solution comes with an identically sized textile “mirror vest” in which each one of the 256 embroidered electrodes is replaced by a CMR-safe fiducial marker (Beekley medical, Bristol, Connecticut, United States). Once the ECGI recording with the electrode vest is complete, the corners of the electrode vest (front and back) are labelled on the patient’s chest with a skin marker pen, and prior to entering the scanner, the mirror vest is applied to these precise locations and fixed there using short strips of tape (3M Transpore, surgical plastic tape). Wearing the mirror vest, the patient undertakes the CMR scan and electrode position co-registration with the torso geometry is achieved.

#### Leads and amplifier

256 ECG leads with snap-on electrode heads provided by g.tec medical engineering GmbH (Schiedlberg, Austria) were used to interface between the graphite connectors in the centre of each dry square textile electrode and a high performance amplifier system, also from g.tec (g.HIamp 256 bundle GT-8016/USBamp GT-0216). g.Hlamp has 256 channels providing a resolution of 8.57 nV and allowing for high oversampling which reduces noise (< 0.5 µV rms 1-30 Hz).

#### ECG signal collection

Prior to CMR, the electrode vest was secured around the participant’s chest and an inflatable gilet (ExotoggⒸ, www.exotogg.com) was worn over the vest to optimize skin–electrode contact. Body surface potentials were recorded from the 256 electrodes for 5 min at rest, in the supine position using the following amplifier settings: no band pass or notch filter; sensitivity range from -10 to + 10mV; sampling frequency of 2400mHz.

Signals were processed for analysis using g.Recorder software (g.tec). In order to minimize electrical interference of the amplified signals all electrical devices were either switched off or removed from the clinic room whenever it was practical to do so. After the recording, the gilet and electrode vest were removed and replaced by the mirror vest to permit CMR scanning. The entire donning/doffing procedure takes < 5 min per participant.

#### CMR scan

All participants were scanned using the same 3 T [200 mT/m/s × 80 mT/m] MRI system (Magnetom Prisma, Siemens Healthineers, Erlangen, Germany) operating VE11C-SP01, with an 18-channel phased-array chest coil and spine array (up to 24-elements) equipped with Gadgetron [37] (Linux box, 16–24 processors). All participants underwent CMR with contrast according to the MyoFit46 with detailed sequence parameters previously described [22]**.** A contiguous free-breathing transaxial set of turbo spin echo (TSE) images using a half-Fourier acquisition single-shot turbo spin echo (HASTE) sequence (flip angle 160°, temporal resolution 251 ms, default field of view 490 mm x 398 mm, prospective triggering, 90–100 contiguous 5 mm slices with no slice gaps) was performed. It was piloted to start above the shoulders and extended down to the mid-abdomen in all participants ensuring complete coverage of all CMR-lucent markers in the mirror vest. To assess cardiac structure and function we acquired conventional breath-held retrospectively electrocardiography (ECG)-gated balanced steady state free precession (bSSFP) cines (flip angle 50°, temporal resolution 30 ms, default field of view 380 mm x 285 mm, retrospective triggering). Left ventricular (LV) volumes and wall thickness were analysed using a validated machine learning pipeline [38].

#### Cardiac segmentation

Heart-torso geometries obtained from the CMR HASTE stack were reconstructed to create individual epicardial meshes using commercially available software (Amira, ThermoFisher, Massachusetts, United States, version 2020.3). The ventricles, atria, aorta and pulmonary arteries were segmented individually, and the resulting epicardial heart mesh was smoothed to generate 2000 individual nodes.

#### Landmark localisation (manual and automated)

**Fig. 2:**
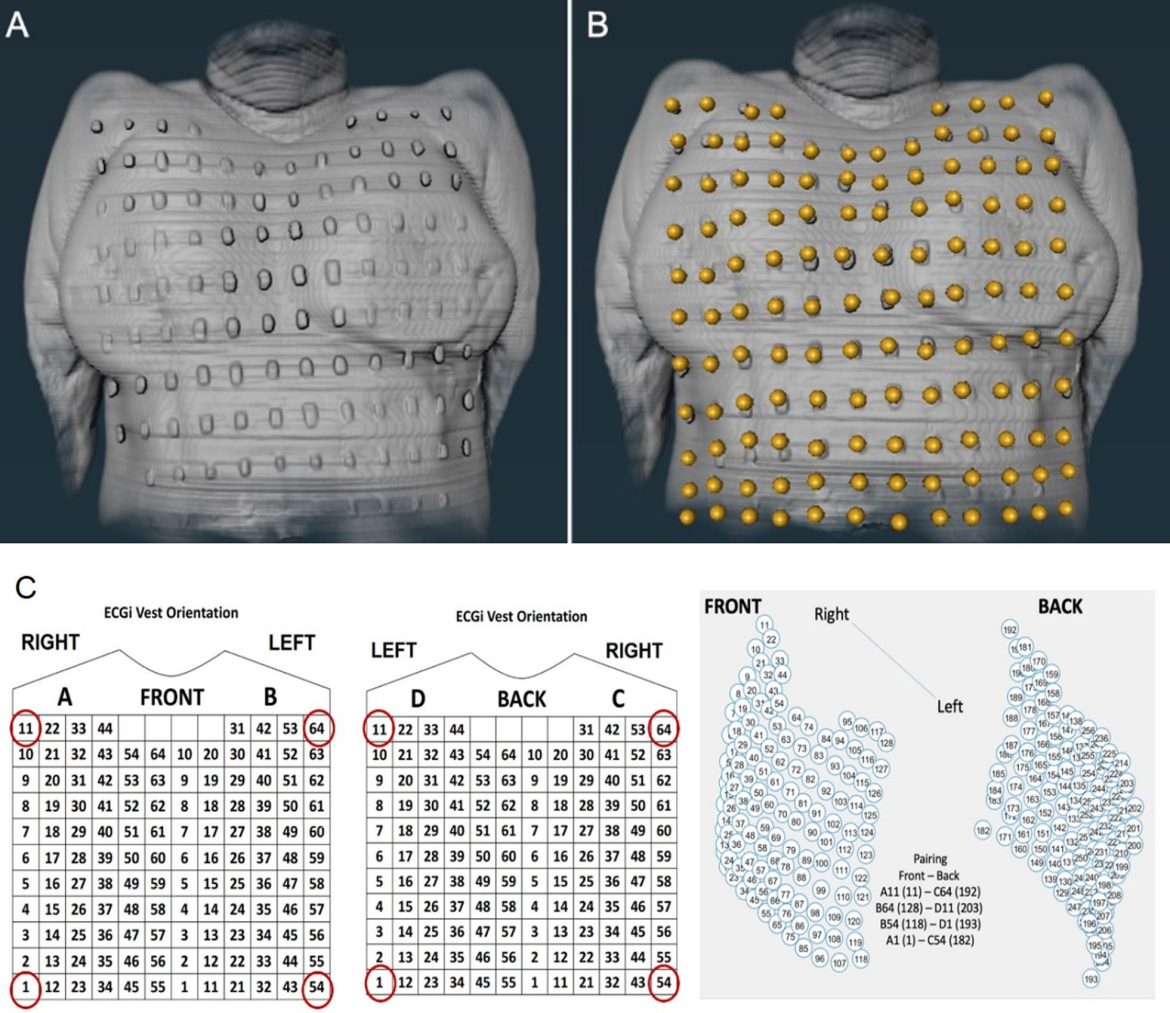
Electrode landmark localisation. **a** Vest fiducial markers clearly visible on the torso surface; **b** Following manual imputation of the landmarks using the software; **c** The ‘virtual’ mirror vest used for the purposes of automated landmark localisation analysis. In order to generate the ‘virtual’ vest, 4 points on each corner of the original vest are manually imputed in order to compute the remaining locations of the other electrodes with accuracy. Abbreviations as per Fig. 1

The location of the 256 fiducial markers on the torso (and corresponding electrode positions) were imputed to create a virtual torso geometry with corresponding lead position data attached as vector code (Fig. 2). A subanalysis in 50 participants was also performed using a ‘virtual’ mirror vest to see if we could speed up the workflow and allow for higher throughput in both data collection and analysis by eliminating the use of the actual mirror vest. A ‘virtual’ mirror vest was reconstructed using in-house Matlab code from four CMR-bright corner markers applied to the participant’s anterior and posterior chest wall to precisely match the corner electrodes of the electrode vest. A computer-generated vest was then generated using a mathematical grid based on the known electrode spacing and taking into account the contour of the torso and back in both males and females. We postulated that this would allow for a more streamlined representation of the electrode landmarks whilst avoiding the need to wear the electrode vest during the CMR scan (Fig. 2).

#### ECGI signal post processing

The pre and post processing steps of the ECG signals collected have been described in detail elsewhere [8, 9, 16, 39]. After ECG data collection, signal averaging of the 256 signals was performed using in-house code for Matlab (MathWorks, Natick, Massachusetts, United States) to enhance the quality of the signals from body surface potentials. Unipolar epicardial electrograms (UEGs) were then reconstructed by solving the ‘inverse solution’ as previously described [9, 21, 39].

These UEGs were then used to compute the activation time (AT) measured as the time of the steepest downslope of the QRS (dV/dt_min_) and the repolarization time (RT) measured as the time of the steepest upslope of the T-wave (dV/dt_max_) for each individual cardiac site. Activation recovery interval, a surrogate of local action potential duration, was measured as ARI = RT-AT. All repolarization parameters were corrected for heart rate (HR) according to Bazzett’s method. Spatial gradients of AT (G_AT_) and RT (G_RT_) were also generated and defined as the average change in value of neighbouring EGM parameters divided by the distance between each EGM location. Global dispersion of AT, RT and ARI (AT_d_, RT_d_ and ARI_d_) were calculated as the difference between the minimum and maximum values within each study participant [20, 21]. Fractionation of the EGM signal (which refers to the number of negative deflections in the QRS thought to be an indicator of abnormal conduction [40]) was also quantified. Once computed, quality control of all parameters was carried out using a semiautomatic pipeline that has been previously validated [41, 42].

### Repeatability studies

To measure the variability in ECGI parameters with repeat measures, intra- and inter-observer variability studies were carried out on 20 consecutively enrolled participants by MW and GJ (each with > 3 years’ experience in CMR and ECGI analysis). These repeatability studies included repeats of all steps in the post-processing pipeline: that is, generation of the heart/torso geometry matrix (cardiac segmentation and electrode landmark imputation), signal averaging and the inverse solution. Analysis was performed using paired cardiac sites whereby the closest corresponding sites were matched across each cardiac mesh. Scan re-scan variability studies were also performed on 8 randomly chosen participants and this involved repeating the ECGI recording and the CMR scan on the same participant at least 3 months after the original scan.

### Statistical methods

Statistical analyses were performed using R [43] (version 4.1.2) and Matlab (MathWorks, Natick, Massachusetts, United States). Data were assessed for normality visually using histograms and through Shapiro–Wilk test and Q-Q plots. Normally distributed continuous variables were expressed as mean ± 1 standard deviation (SD); non-normally distributed continuous variables as median and interquartile range (IQR); categorical variables as counts and percentages.

Repeatability was appraised according to the following parameters due to the non-gaussian nature of the data: Pearson’s correlation coefficient measuring the similarity of UEG morphology across all cardiac sites (*r*UEG); rank-based Spearman’s correlation coefficient to determine similarity of the AT, RT and ARI sequence per participant (*r*_s_AT, *r*_s_RT, *r*_s_ARI); absolute difference in AT, RT and ARI between all cardiac sites (∆AT, ∆RT, ∆ARI); intra-class correlation coefficient (ICC), 2-way random effects without interactions [44] measuring the similarity of AT, RT and ARI sequence per participant (iccAT, iccRT, iccARI) and the similarity of UEG morphology (iccUEG). UEG similarity was assessed by comparing the entire waveform between paired UEGs (measured from the corresponding cardiac sites). Bland–Altman plots were also used to further assess agreement in intra and inter-observer repeatability analyses. Because between-observer differences were non-normally distributed, a non-parametric approach was adopted to estimate limits of agreement [45] with the median used to assess bias and the 5^th^ and 95^th^ percentiles representing the lower and upper limits, respectively. The analysis was conducted per case considering all paired cardiac sites (n = 12′937, same across all participants), and confidence intervals (CI) for global limits of agreement were defined as the interquartile ranges of their distribution**.** Scan re-scan variability was analysed using spearman’s correlation coefficients and ICC of the mean AT, RT and ARI across all cardiac sites but UEG morphology similarity was not assessed for each of the 12,000 + points due to misalignment of the cardiac meshes generated across the 2 scans.

To test for differences between younger and older age participants, an independent sample t-test was used for normally distributed data, a Mann–Whitney U test for non-normally distributed data and a Chi squared test for categorical data. Correlations between 12-lead ECG and ECGI parameters was carried out using Spearman’s test due to their non-parametric nature. Missing data were dealt with via exclusion from the final statistical analysis. The valve plane was manually removed from all analysis (including repeatability studies) due high number of anomalous EP variability across these regions, which is in keeping with previously validated ECGI methodology. Statistical significance was defined as a *p* value < 0.05 and presented alongside either SD or CI as appropriate.

## Results

### Baseline characteristics

A total of 77 participants were recruited consisting of 50 older persons from the MyoFit46 study and 27 young healthy volunteers. Participant characteristics, CMR and ECGI parameters are presented in Table 1. Males were more frequent in the older than the younger sample (males, 52% vs 35%, *p* = 0.23) but there were negligible differences in BSA (1.82 vs 1.79, *p* = 0.57) or heart rate. (63.4 vs 68.3 bpm, *p* = 0.08). A summary of the past medical and medication history of the older age sample is also presented in Table 1**.** CMR parameters were similar between the two groups. A summary of 12-lead ECG data is also presented in Table 1**.** QRS duration and QTc time were longer in older compared to younger participants (99.97 ms vs 93.60 ms, *p* = 0.03 and 421.52 ms vs 409.60 ms, *p* = 0.001 respectively).

**Table 1:** Baseline characteristics

Group	Younger age participants (HV) *n* = 27	Older age participants (MyoFit46) *n* = 50	*p* value*
Demographics			
Age, years	38.9 ± 8.5	77.0 ± 0.1	** < 0.001**
Male, n	9 [35]	26 [51]	0.23
BSA, m^2^	1.79 ± 0.25	1.82 ± 0.17	0.57
Heart rate, bpm	68 ± 11	63 ± 9.7	**0.08**
Past medical history			
T2DM	0 (0)	2 (4)	**NA**
HTN	0 (0)	15 (30)	**NA**
Chol	0 (0)	18 (36)	**NA**
MI	0 (0)	1 (2)	**NA**
IHD	0 (0)	3 (6)	**NA**
AF	0 (0)	0 (0)	**NA**
AB	0 (0)	2 (4)	**NA**
ACEi	0 (0)	11 (22)	**NA**
BB	0 (0)	4 (4)	**NA**
CCB	0 (0)	6 (6)	**NA**
CMR parameters			
LAVi,ml	21 ± 3.9	22 ± 6.8	0.28
LVEDVi, ml	81 (12)	75 (12)	0.21
MAPSE, mm	18 ± 4.3	15 ± 2.9	**0.002**
LVEF, %	72 (7.2)	72 (7.8)	0.28
LVSVi, ml	41 (15)	40 (15)	0.71
RVEDVi, ml	99 (19)	83 (21)	** < 0.001**
TAPSE, mm	23 ± 3.7	23 ± 3.9	0.58
RVEF, %	58 ± 4.3	60 ± 5.2	0.068
12-lead ECG			
RR, ms	929 ± 139	941 ± 133	0.75
PR, ms	160 ± 23	174 ± 37	0.06
QRS, ms	94 ± 8.6	100 ± 15	**0.03**
QTc, ms	410 ± 20	422 ± 26	**0.001**
ECGI parameters			
Amp, mV	1.41 ± 0.34	1.41 ± 0.37	0.97
AT, ms	41 (10)	44 (7.7)	0.30
RT, ms	289 ± 20	296 ± 21	**0.002**
ARI, ms	235 ± 18	250 ± 21	**0.002**
AT_d_, ms	11 (3.5)	13.6 (4.4)	**0.01**
RT_d_, ms	28 (8.9)	31 (13)	0.09
ARI_d_, ms	32 ± 9.0	37 ± 12	**0.04**
G_AT_, ms/mm	0.34 (0.12)	0.40 (0.13)	**0.01**
G_RT_, ms/mm	0.77 (0.23)	0.92 (0.40)	**0.03**
G_ARI_, ms/mm	0.92 ± 0.27	1.12 ± 0.40	**0.01**
Frac, n	1.004 (0.01)	1.003 (0.01)	0.88

Statistics are presented as mean ± SD, median (IQR) or n [%]

Significant *p* values are highlighted in bold

*Amp* amplitude, *AB* alpha-blocker, *ACEi* angiotensin converting enzyme inhibitor, *AT* activation time, *ARI* activation recovery interval, *BB* beta-blocker, *BSA* body surface area, *CCB* calcium channel blocker, *Chol* hypercholesterolaemia, *HTM* hypertension, *ECG* electrocardiogram, *Frac* fractionation, *G*_*AT,*_* G*_*RT,*_* G*_*ARI*_ spatial gradient of AT, RT and ARI respectively, *IHD* ischaemic heart disease, *LAVi* BSA-indexed left atrial volume, *LVEF* left ventricular ejection fraction, *LVEDVi* BSA-indexed left ventricular end diastolic volume, *LVSVi* BSA-indexed left ventricular systolic volume, *MAPSE* mitral annular plane systolic excursion, *MI* myocardial infarction, *RVEDVi* BSA-indexed right ventricular end diastolic volume, *RVEF* right ventricular ejection fraction; *RT* repolarization time, *TAPSE* tricuspid annular plane systolic excursion, *T2DM* type 2 diabetes, *AT*_*d*_*, RT*_*d*_*, ARI*_*d*_ global dispersion of AT, RT and ARI respectively

^*^Differences between younger and older participants were measured using unpaired t-test, Mann–Whitney U or Chi-squared test as appropriate

### Feasibility and time to deploy

**Fig. 3:**
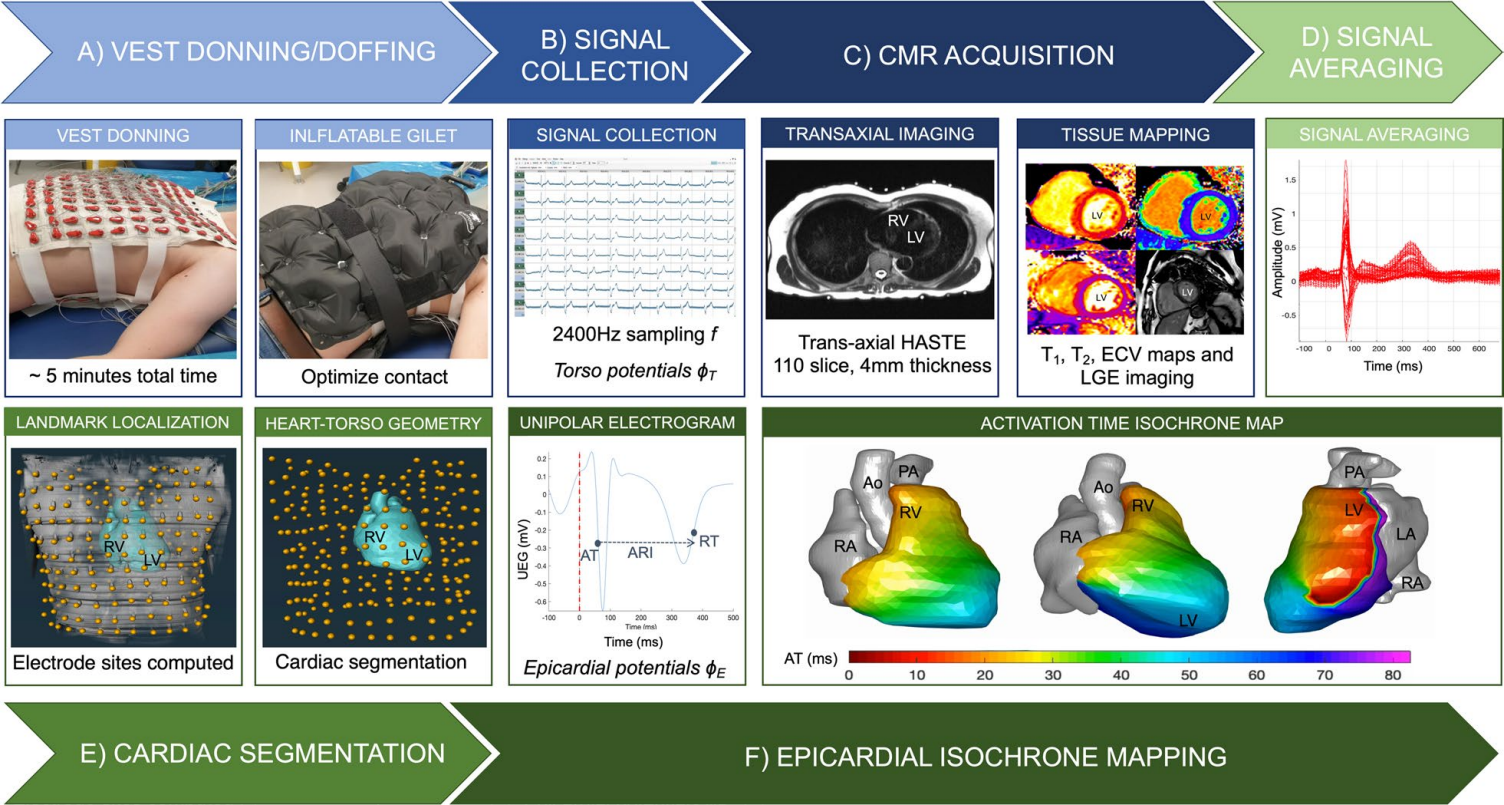
Step by step CMR-ECGI workflow. **a** The participant lies in the supine position for a 5-min recording at rest and an inflatable gilet is worn over the electrode vest to ensure good skin–electrode contact. **b** 256 ECG signals (torso potentials ϕ_T_) are collected from the dry textile electrodes and amplified using the g.HIamp at a sampling frequency of 2400 Hz and processed using g.Recorder. **c** CMR scanning is performed at 3T to include T1 mapping, T2 mapping, LGE and ECV and transaxial HASTE used for cardiac segmentation and landmark localisation. **d** ECG signals are post processed using in-house Matlab software. **e** Heart-torso geometry (A) is generated from the HASTE via the reconstruction of individual cardiac meshes and imputation of the electrode markers using Amira software. **f** Heart-torso geometry (A) is combined with the torso potentials (ϕ_T_) to solve the ‘inverse solution’ of ECGI (ϕ_T_ = Aϕ_E_) to reconstruct epicardial potentials (ϕ_E_) at 1000 individual cardiac sites which are used to compute AT, RT and ARI per site and generate isochrone maps of cardiac excitation. In this example of a 75-year-old participant the earliest site of activation is in the posterolateral lateral wall of the LV. *Ao* aorta, *ARI* activation recovery interval, *ECV* extracellular volume, *F* frequency, *LA* left atria, *LGE* late gadolinium enhancement, *LV* left ventricle, *mV* millivolts, *ms* milliseconds, *PA* pulmonary artery, *RA* right ventricle, *RV* right ventricle, *UEG* unipolar electrogram. Other abbreviations as in Fig. 1

**Fig. 4:**
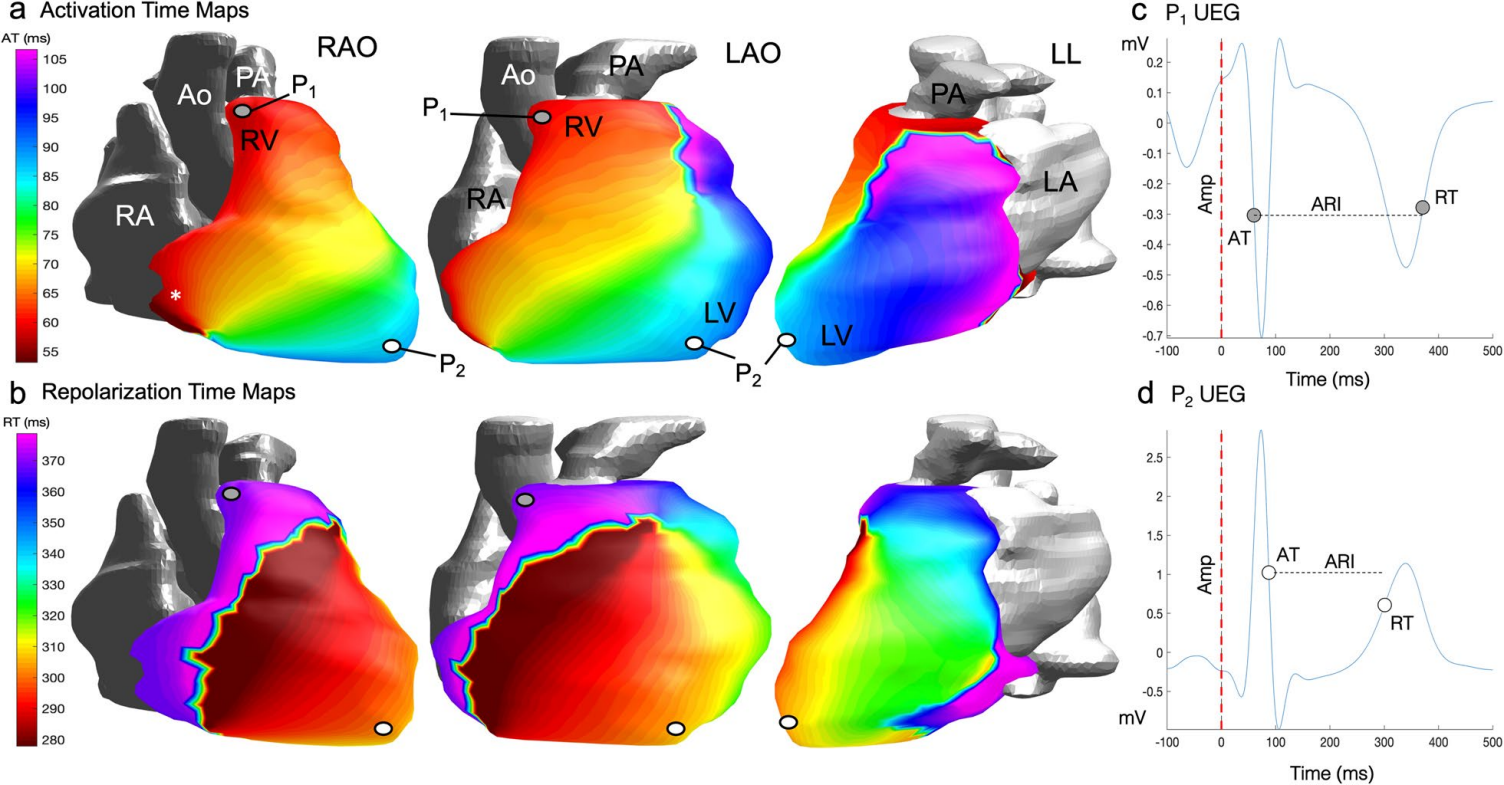
Epicardial isochrone maps. **a** AT maps in a healthy 75 year old male participant from the MyoFit46 cohort oriented here to mimic the 3 classic angiographic views in normal sinus rhythm. The earliest epicardial breakthrough (*) occurs in the basal RV in this participant. **b** RT maps in the same participant showing the wave of epicardial repolarization travelling from apex to base. **c** Reconstructed epicardial UEG for P_1_ which represents one node out of 1000 generated for individual cardiac sites—it highlights a negative QRS and T wave signal at the cardiac base. **d** Reconstructed epicardial UEG for P_2_ highlighting a positive QRS and T-wave signal at the cardiac apex. Each UEG is used to compute individual AT, RT, ARI and amplitudes per node which can then be averaged across all cardiac sites for the purposes of analysis. *Amp* amplitude, *LAO* left anterior oblique, *LL* left lateral, *P*_*1*_ point 1, *P*_*2*_ point 2, *RAO* right anterior oblique. Other abbreviations as in Fig. 1

All ECGI recordings were completed without complications with a total time of < 10 min per participant (including 5-min recording time). Total post-processing time took approximately ~ 15 min per participant (this includes HASTE segmentation, signal averaging and landmark localisation. The same vest was used on all participants following rigorous cleaning protocols between studies. Epicardial isochrone maps were successfully constructed in every participant without any failures using the post-processing steps outlined and are summarised in Fig. 3. In most cases the first breakthrough occurred in the basal RV and the wave of activation spread to the apex but alternative sites of breakthrough were also observed which is in keeping with previously validated work on healthy human subjects [9, 46, 52]. A representative example of the process of normal activation and recovery using our CMR-ECGI solution is illustrated in Fig. 4.

### Repeatability studies

CMR-ECGI repeatability results are presented in Table 2**.** In the 20 cases that underwent tests for intra- and inter-observer variability, the median correlation coefficients measuring the similarity of UEGs across all paired cardiac sites (*r*_s_UEG) was consistently strong (intra-observer *r*_s_UEG: 0.99 [IQR 0.97, 0.98] and iccUEG: 0.93 [IQR 0.83, 0.98]; inter-observer *r*_s_UEG: 0.98 [IQR 0.94, 1.00] and iccUEG: 0.92 [IQR 0.81, 0.97], n = 12,937).

**Table 2:** Repeatability tests results

Repeatability parameter	Intra-observer	Inter-observer
*r*_s_UEG	0.99 (0.97–1.00)	0.98 (0.94–1.00)
*r*_s_AT	0.88 (0.79–0.93)	0.86 (− .70–0.88)
*r*_s_RT	0.83 (0.81–0.89)	0.76 (0.72–0.85)
*r*_s_ARI	0.80 (0.79–0.87)	0.75 (0.71–0.81)
iccUEG	0.93 (0.83–0.98)	0.92 (0.81–0.97)
iccAT	0.93 (0.85–0.96)	0.88 (0.75–0.90)
iccRT	0.90 (0.87–0.92)	0.83 (0.80–0.91)
iccARI	0.90 (0.85–0.93)	0.85 (0.81–0.90)
∆AT, ms	1.25 (0.42–2.50)	1.25 (0.42–3.33)
∆RT, ms	2.08 (0.83–4.58)	2.08 (0.83–6.25)
∆ARI, ms	2.08 (0.83–5.00)	2.50 (0.83–7.50)
∆AT, %	0.91 (0.34–2.04)	1.15 (0.43–2.64)
∆RT, %	0.54 (0.21–1.24)	0.62 (0.23–1.75)
∆ARI, %	0.84 (0.34–2.11)	1.13 (0.41–3.28)

All *p* values (not shown) are statistically significant

Data are presented as median (IQR)

*icc* inter-class correlation coefficient 2-way random effects without interactions

*r*_*s*_*UEG* average spearman’s correlation coefficient across each individual cardiac site (n = 12′937), *r*_*s*_*AT, r*_*s*_*RT and* *r*_*s*_*ARI* average spearman’s correlation coefficient measuring the similarity of the activation, repolarization and ARI sequence per participant (n = 20 in each group), *iccUEG* average icc across each cardiac site (n = 12′937); iccAT, iccRT and *iccARI* average icc measuring the similarity of the activation, repolarization and ARI sequence per participant (n = 20 in each group); ∆AT, ∆RT and *∆ARI* absolute difference in AT, RT and ARI across each individual cardiac site (n = 12′937) as presented in ms and %; *UEG* unipolar electrogram; Other abbreviations as in Tables 1

**Fig. 5:**
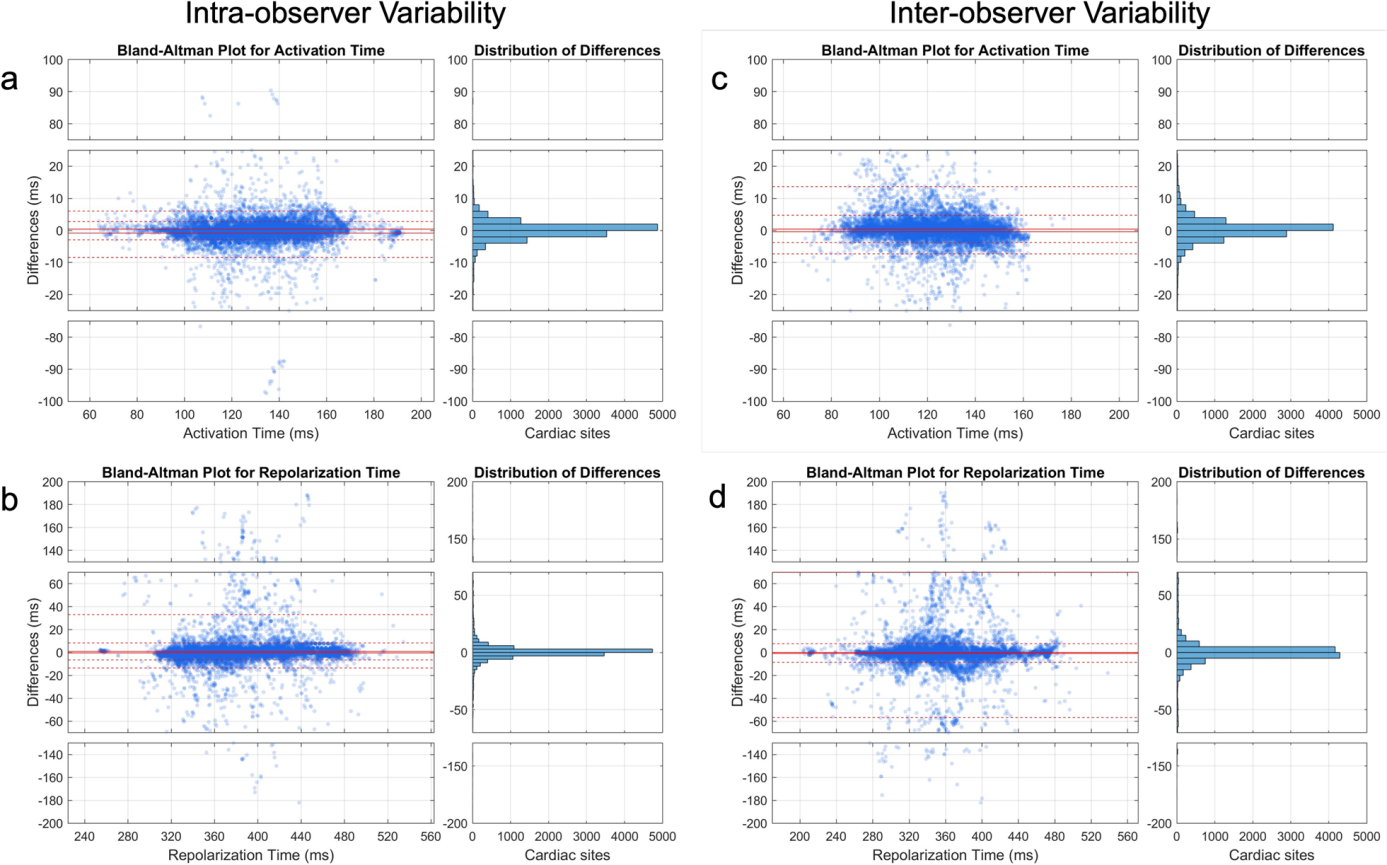
Bland–Altman plots and histogram for distribution of differences measuring the similarity across observers. **a** AT sequence similarity for intra-observer variability; **b** RT sequence similarity for intra-observer variability; **c** AT sequence similarity for inter-observer variability; **d** RT sequence similarity for inter-observer variability. Because between-observer differences were non-normally distributed, a non-parametric approach was adopted to estimate limits of agreement with the median used to assess bias and the 5th and 95th percentiles representing the lower and upper limits, respectively. The analysis was conducted for each case (considering all paired cardiac sites) across observers (n = 12,937 per case, represented by each dot). Confidence intervals for global limits of agreement were defined as the interquartile range of their distribution. Abbreviations as in Fig. 1

The median correlation coefficients measuring the similarity of the electrical sequence with respect to AT RT and ARI per participant were also strong (intra-observer *r*_s_AT: 0.88 [0.79, 0.93] and iccAT: 0.93 [0.85, 0.96]; *r*_s_RT: 0.83 [0.81, 0.89] and iccRT: 0.90 [0.87, 0.92]; *r*_s_ARI: 0.80 [0.79, 0.87] and iccARI: 0.90 [0.85, 0.93]; inter-observer *r*_s_AT: 0.86 [0.70, 0.88] and iccAT: 0.92 [0.81, 0.97]; *r*_s_RT: 0.76 [0.72–0.85] and iccRT: 0.83 [0.80, 0.91]; *r*_s_ARI: 0.75 [0.71, 0.81] and iccARI: 0.85 [0.81, 0.90]). Bland–Altman plots and histograms for distribution of differences measuring the similarity of AT and RT sequence per participant covering paired cardiac sites across observers can be found in Fig. 5**.**

The median absolute differences across all cardiac sites (n = 12,937) with respect to AT (∆AT), RT (∆RT) and ARI(∆ARI) were consistently small (intra-observer ∆AT:1.25 ms [IQR: 0.42, 2.50 ms]; ∆RT: 2.08 ms [0.83, 4.58 ms]; ∆ARI: 2.08 ms [0.83, 5.00 ms] and inter-observer ∆AT: 1.25 ms [0.42, 3.33 ms]; ∆RT: 2.08 ms [0.83, 6.25 ms]; ∆ARI: 2.50 ms [0.83, 7.50 ms]).

**Fig. 6:**
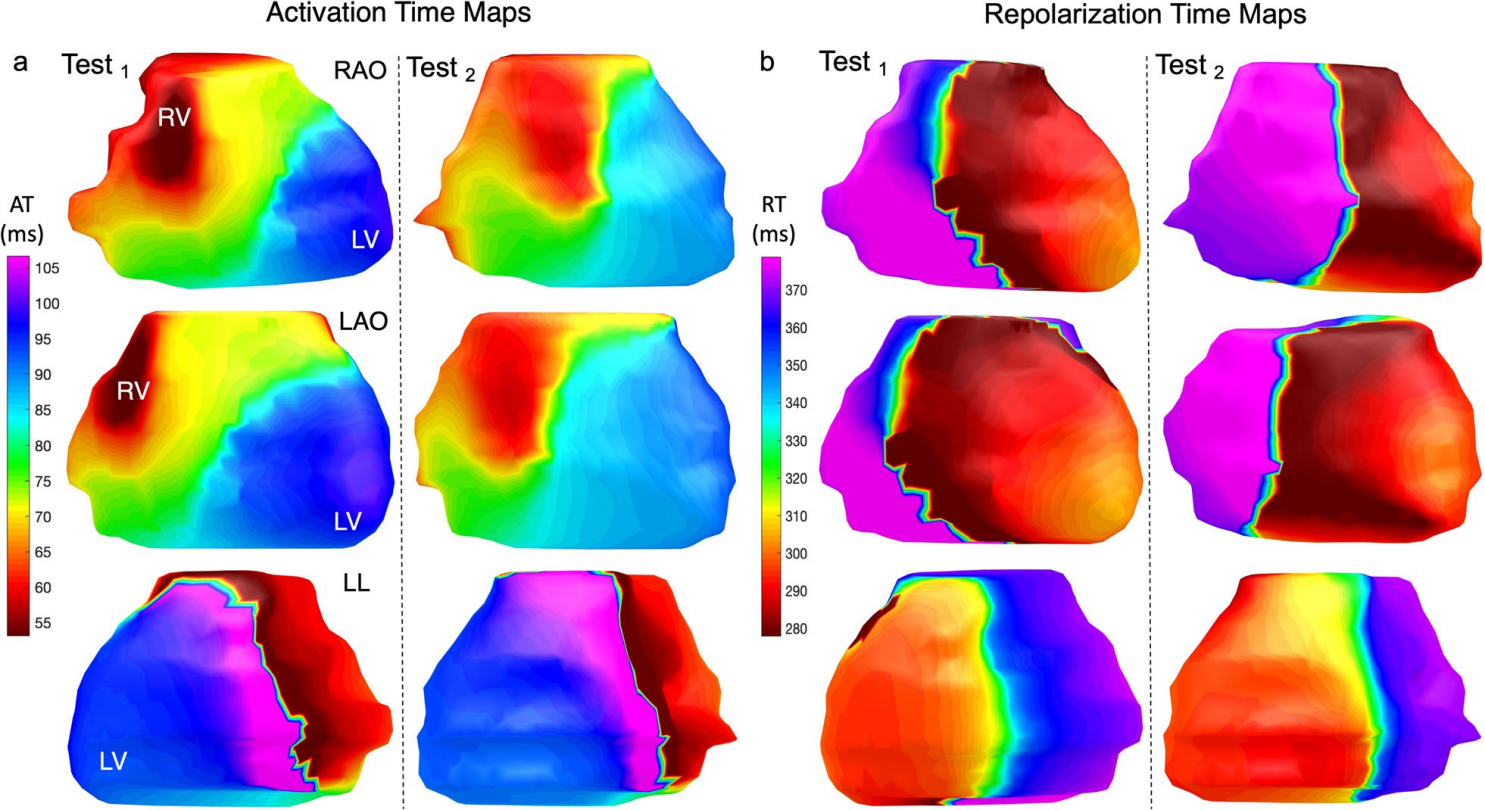
Consistency of epicardial isochrone maps as shown by the scan re-scan repeatability studies. **a** AT map of a healthy 37-year old male participant shown in the 3 classic angiographic views. **b** Corresponding RT map of the same participant. Averaged ECGI parameters across all cardiac nodes showed excellent spatial correlation across all test/re-test reproducibility studies as described in this manuscript. Abbreviations as in Figs. 1 and 2

**Fig. 7:**
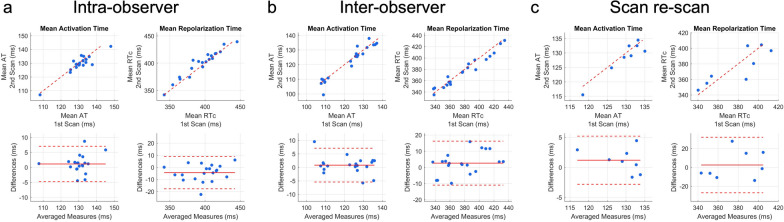
Intraclass correlation coefficient and Bland–Altman plots measuring the similarity of global mean AT and RT. Top panels show the intra-class correlation coefficient plots while bottom panels show the Bland–Altman plots comparing mean AT and RT for intra-observer tests (r = 0.95 for AT and 0.98 for RT); inter-observer tests (r = 0.97 for AT and 0.98 for RT); and scan re-scan studies (r = 0.96 for AT and 0.76 for RT). RTc Bazzetts heart rate corrected repolarization time; Abbreviations as in previous figures

Scan re-scan studies showed high reliability between mean AT, RT and ARI computed across all epicardial sites (*r*_s_ = 0.81; *r*_s_ = 0.86, ARI *r*_s_ = 0.93, respectively and *r*_icc_ = 0.96 m, *r*_icc_ = 0.76, *r*_icc_ = 0.78 respectively). Visually there was consistency across all scan re-scan epicardial isochrone maps and an exemplar pair is shown in Fig. 6**.** ICC and Bland–Altman plots measuring the similarity of the global mean AT and RT across intra-observer, inter-observer and scan re-scan reproducibility studies can be found in Fig. 7**.**

### Virtual mirror vest

Results for the subanalysis using only corner markers and virtual mirror vest relative to manual imputation of data from the mirror vest (n = 50) are presented in Additional file 3: Table S1. The median correlation coefficients measuring the similarity of UEGs across all paired cardiac sites (*r*_s_UEG) was strong (*r*_s_UEG: 0.99 [IQR 0.98, 1.00] and iccUEG: 0.99 [IQR 0.97, 1.00]). The median correlation coefficients measuring the similarity of the electrical sequence with respect to AT, RT and ARI per participant were also strong (*r*_s_AT: 0.92 [0.86, 0.96] and iccAT: 0.95 [0.92, 0.97]; *r*_s_RT: 0.86 [0.82, 0.91] and iccRT: 0.92 [0.87, 0.95]; *r*_s_ARI: 0.84 [0.79, 0.90] and iccARI: 0.92 [0.86, 0.95]). The median absolute differences across all cardiac sites (n = 12,937 nodes) with respect to AT (∆AT), RT (∆RT) and ARI(∆ARI) were consistently small (∆AT:0.83 ms [IQR: 0.42, 2.08 ms]; ∆RT: 1.25 ms [0.41, 3.33 ms]; ∆ARI: 1.25 ms [0.42, 3.75 ms]). Bland–Altman plots for distribution of differences measuring the similarity of AT and RT sequence per participant are provided in Additional file 1: Fig. S1 while ICC and Bland–Altman plots measuring similarities of global mean AT and RT can be found in Additional file 2: Fig. S2.

### Comparisons with 12-lead ECG data

A sub-analysis was carried out looking specifically into the associations with 12-lead ECG data and ECGI derived parameters. PR interval correlated with AT and G_RT_ (r_s_ -0.27, p = 0.028; r_s_ 0.26, p = 0.032 respectively). QRS interval correlated with RT (r_s_ 0.28, p = 0.02). QT interval correlated with RT and ARI (r_s_ 0.43, p = 0.001; r_s_ 0.49, p < 0.001 respectively).

### Differences in ECGI parameters between young and old people

**Fig. 8:**
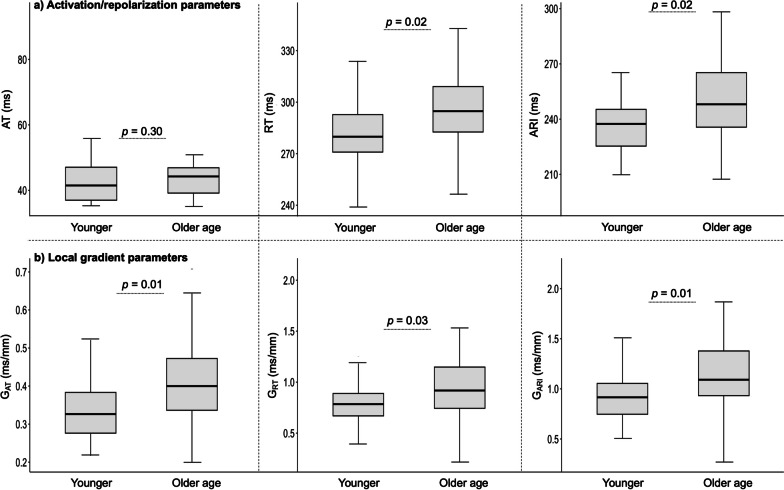
Comparison of ECGI parameters between young and older age participants. Central line represents the median, edges of the boxes are the first and third quartiles and the whiskers extend to the extreme ends of the data not considered to be outliers. *G*_*AT*_ local gradient of activation time, *G*_*RT*_ local gradient of repolarization time, *G*_*ARI*_ local gradients of activation recovery interval. Other abbreviations as in Figs. 1 and 2

As presented in Fig. 8**,** EP parameters measured by CMR-ECGI were longer in the older compared to the younger group. Older age participants had longer RT (296.8 vs 289.3 ms, *p* = 0.002) and ARI (249.8 vs 235.1 ms, *p* = 0.002) when compared to younger participants. AT tended to be longer in the older age participants however this did not reach statistical significance (44.3 vs 41.5 ms, *p* = 0.30). Local spatial gradients (G) of the EP parameters were larger in the older compared to the younger cohort (for G_AT_: 0.40 vs 0.34 ms/mm, *p* = 0,01, G_RT_: 0.92 vs 0.77 ms/mm, *p* = 0.03 and G_ARI_: 1.12 vs 0.92 ms/mm, *p* = 0.01). Local dispersions of EP parameters were similarly increased in the older compared to the younger cohort for AT_d_ (13.62 vs 10.68 ms, *p* = 0.01) and ARI_d_ (36.98 vs 31.76 ms, *p* = 0.04) but not for RT_d_ (30.78 vs 27.99 ms, *p* = 0.09). Sub-analysis confirmed that these age-related changes were independent of sex even in the older group which was male predominant.

## Discussion

In this study, we describe the first high-throughput, reusable ECGI vest for seamless use with CMR. It is the largest ECGI-CMR integrated study to date and our methods used are shown to be reliable after comprehensive repeatability experiments, all with excellent results. It is also the first time that these ECGI pipelines have been tested for their ability to detect small differences in age-related EP parameters. Through integration with advanced post-processing techniques, the CMR-ECGI vest allows for detailed non-invasive collection of EP data to generate real-time panoramic maps of cardiac activation and recovery patterns under complete physiological conditions. This scalable approach holds the potential to provide valuable insights into the arrhythmogenic consequences of myocardial structural changes as detected by CMR. We have shown feasibility of our methodology in a group of older age patients. In addition, we have developed an efficient post processing pipeline including a novel method for automated landmark localisation to provide sufficiently faithful reconstruction of torso geometry which could allow for a more streamline analysis approach.

Our vest was designed to conform to a range of male and female adult body sizes. However, it could also be bespoke fabricated to suit younger persons (to carry out CMR-ECGI in children for example). All our study participants fitted comfortably inside the 60 cm diameter 3T scan bore of our Siemens magnet, indicating that this vest size is appropriate for any adult with a torso circumference of < 180 cm. In theory a larger vest could also be designed but it is unlikely that subjects with a chest circumference > 180 cm will fit comfortably inside a 3T scanner bore.

Through use of our vest, we have found that older age persons have prolonged RTs and ARIs when compared to younger persons. Potential mechanisms underlying this observation could be due to age-related changes in cardiac ion channels and calcium handling that would lead to alterations in the action potential duration and recovery [47]. The fact that these EP parameters were significantly longer in older participants in the absence of differences in AT, suggests that abnormal patterns of repolarization may be independent of action potential propagation. It also indicates that conduction and repolarization processes are highly complex and likely to be modulated at the level of the myocytes and contributed to by the spatial distribution of abnormal cardiac tissue. Furthermore, our results also showed that ageing associated with increased local gradients of AT, RT and ARI which implies increased differences/variations in EP potentials across cardiac regions [48]. This suggests the presence of abnormal electrical conduction and recovery at the tissue level which could be mediated by age-related alterations in gap junctions (number and size) and reduced cellular uncoupling because of cardiac remodelling or localized myocardial fibrosis [49–51].

The main aims of this prospective study were technical development and feasibility of the pipeline. The inverse solution applied to CMR-ECGI has already been previously validated *ex vivo* [11, 12], in vivo animal studies [13, 14] and invasively in humans [15, 52] and such experiments were not repeated here. Prolonged EP parameters generated from our pipeline such as RT and ARI are known to provide substrates for re-entrant circuits and have been previously shown to be associated with arrhythmogenesis and SCD in previous studies [53, 54]. This future work exploring the correlation between spatially co-registered EP metrics and CMR biomarkers of diffuse inflammation/fibrosis is planned for the MyoFit46 study once cohort recruitment is complete. We are currently working on refining our analysis pipeline to include automated segmentation of the LV ECGI data according to (American Heart Association) AHA segments to permit this.

Currently available ECGI solutions are not scalable because of their reliance on radiation-positive CT, high cost, single use design and time-consuming or cumbersome set ups [20, 21]. Being washable and reusable on several hundreds of patients, our proposed CMR-ECGI solution is more affordable than existing technologies and makes for a more practical, scalable, and sustainable option for the use in both research and clinical applications. Through seamless integration with CMR we aim to bridge the current knowledge gap between EP variables and multi-parametric CMR biomarkers and we believe that our solution has the potential to enhance several clinical ECGI use cases, with some examples being: a) promoting personalized SCD risk stratification beyond conventional criteria and guide the need for ICD [55]; b) facilitating the study of arrythmia in vivo (atrial and ventricular) to provide guided targets for pharmacological or invasive catheter ablation treatments [15, 56]; and c) predicting cardiac resynchronization therapy response by guiding lead placement to improve the chances of ventricular synchronization [57].

### Limitations

While our sample size of n = 77 is the largest integrated CMR-ECGI study to date, the MyoFit46 study is scheduled to complete recruitment of its target 550 participants by the end of 2023 when further large-scale CMR-ECGI insights will become available. Once this is completed, we will be able to explore a wider range of pathological processes as well as BSA range (which was limited to 1.79–1.82 in this study). Another limitation to note is that focal measures of myocardial tissue characterisation were not assessed in this study due to its feasibility nature however this does limit any clinical assumptions based on focal EP and CMR parameters which will need to be explored in future work. Exclusion of septal segments in ECGI also limits the generalisability towards clinical adoption in patients with septal infarct/fibrosis. Using the well-validated inverse solution algorithm by Rudy et al., we also noted instances of isochrone crowding in regions of bipolar amplitude voltages. This is a known limitation of all current ECGI inverse solution algorithms, but some groups are working on possible solutions that might soon be available for wider testing [58].

Our results are consistent with what has previously been described on the study of ECGI in the context of both CT [8, 9] and CMR [20, 21]. Previous studies have reported conflicting findings when comparing commercially available ECGI systems with invasively measured action potentials [59], however our chosen inverse solution is one of the most extensively validated in both humans and animal models as highlighted previously. We have shown excellent intra- and inter-observer repeatability of our workflow with only limited residual variation between observers (specifically 3–6% of data showed > 25% regional differences) likely because of mis-labelled UEGs that escaped semi-automated and manual correction. This is a known limitation of all current ECGI post-processing pipelines and one that we and others are working to improve. Importantly, the similarly of UEG morphology was high which highlights the consistency of our ECGI pipeline in this context. Repeatability of a commercially available ECGI system has been studied previously but never to this extent or across such a large cohort of younger and older persons, with such positive results.

## Conclusions

High-throughput CMR-ECGI with a reusable vest opens the door to non-invasive and radiation-free whole-heart panoramic EP mapping suitable for healthcare and large-scale population research studies. CMR-ECGI could provide novel insights into the complex mechanisms of arrythmia potentially paving the way for more personalized risk stratification in patients with heart muscle disease.

### Supplementary Information

**Additional file 1: Figure S1.** Bland–Altman plots and histogram for distribution of differences across automatic vs manually generated mirror vests. **a** AT sequence similarity; **b** RT sequence similarity. A non-parametric approach was adopted to estimate limits of agreement with the median used to assess bias and the 5th and 95th percentiles representing the lower and upper limits, respectively. The analysis was conducted for each case (considering all paired cardiac sites) represented by each dot. Confidence intervals for global limits of agreement were defined as the interquartile range of their distribution. Abbreviations as in Fig. 1

**Additional file 2: Figure S2.** Intraclass correlation coefficient and Bland–Altman plots for automatic vs manually generated mirror vests for landmark localisation. Top panels show the intra-class correlation coefficient plots while bottom panels show the Bland–Altman plots comparing global mean AT (ICC 0.95 [0.92–0.97] and RT (ICC 0.92 [0.87–0.95]) across automatically generated mirror vest for landmark localisation vs manual imputation (n = 50). Abbreviations as in previous Figs. 1 and 7.

**Additional file 3: Table S1.** Validation of ‘virtual’ automatically generated mirror vest for landmark localisation vs manual imputation (n = 50)

## Data Availability

GC has full access to all the data reported in this study and takes responsibility for its integrity and the data analysis. The datasets used/or analysed during this study are available from the corresponding author on reasonable request. NSHD data are available here: https://www.nshd.mrc.ac.uk/data and the statistical analysis code is available on GitHub: https://github.com/gcaptur/CMR-ECGi.

## Abbreviations

Ag/AgCl: Silver/silver chloride
AHA: American Heart Association
ARI: Activation repolarization interval
AT: Activation time
BSA: Body surface area
bSSFP: Balanced steady state free precession
CI: Confidence interval
CMR: Cardiovascular magnetic resonance
CRT: Cardiac resynchronization therapy
CT: Computerized tomography
d: Local dispersions.
ECG: Electrocardiogram
ECGI: Electrocardiographic imaging
ENSAIT: École Nationale Supérieure des Arts et Industries
EP: Electrophysiology
G: Local gradients
HASTE: Half-Fourier acquisition single-shot turbo spin echo
HR: Heart rate
ICD: Implantable cardioverter defibrillator
LV: Left ventricle
NCT: National Clinical Trials
NSHD: National Survey of Health and Development
REC: Research Ethics Committee
RT: Repolarization time
RV: Right ventricle
SCD: Sudden cardiac death
SD: Standard deviation
TSE: Turbo spin echo
T1MES: T1 mapping and ECV standardization
UEG: Unipolar epicardial electrogram
VT: Ventricular tachycardia
