# Supplementary figures and images for: Technical development and feasibility of a reusable vest to integrate cardiovascular magnetic resonance with electrocardiographic imaging
Source: J Cardiovasc Magn Reson. 2023 Dec 4;25:73. doi: 10.1186/s12968-023-00980-7 (PMC10694972; doi:10.1186/s12968-023-00980-7)

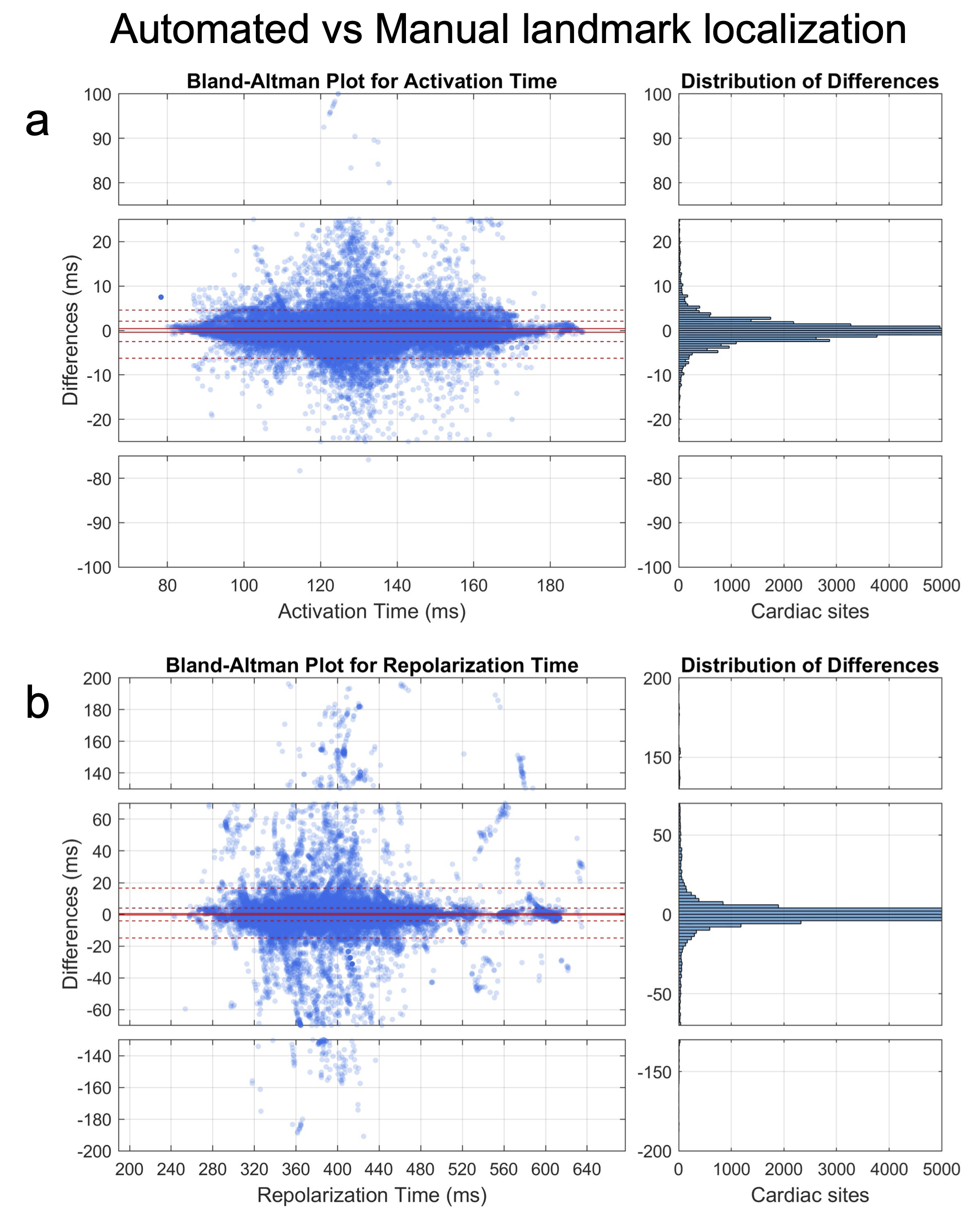

Supplement: Supplementary file 1 — Additional file 1: Figure S1. Bland–Altman plots and histogram for distribution of differences across automatic vs manually generated mirror vests. a AT sequence similarity; b RT sequence similarity. A non-parametric approach was adopted to estimate limits of agreement with the median used to assess bias and the 5th and 95th percentiles representing the lower and upper limits, respectively. The analysis was conducted for each case (considering all paired cardiac sites) represented by each dot. Confidence intervals for global limits of agreement were defined as the interquartile range of their distribution. Abbreviations as in Fig. 1 [file 12968_2023_980_MOESM1_ESM.png]

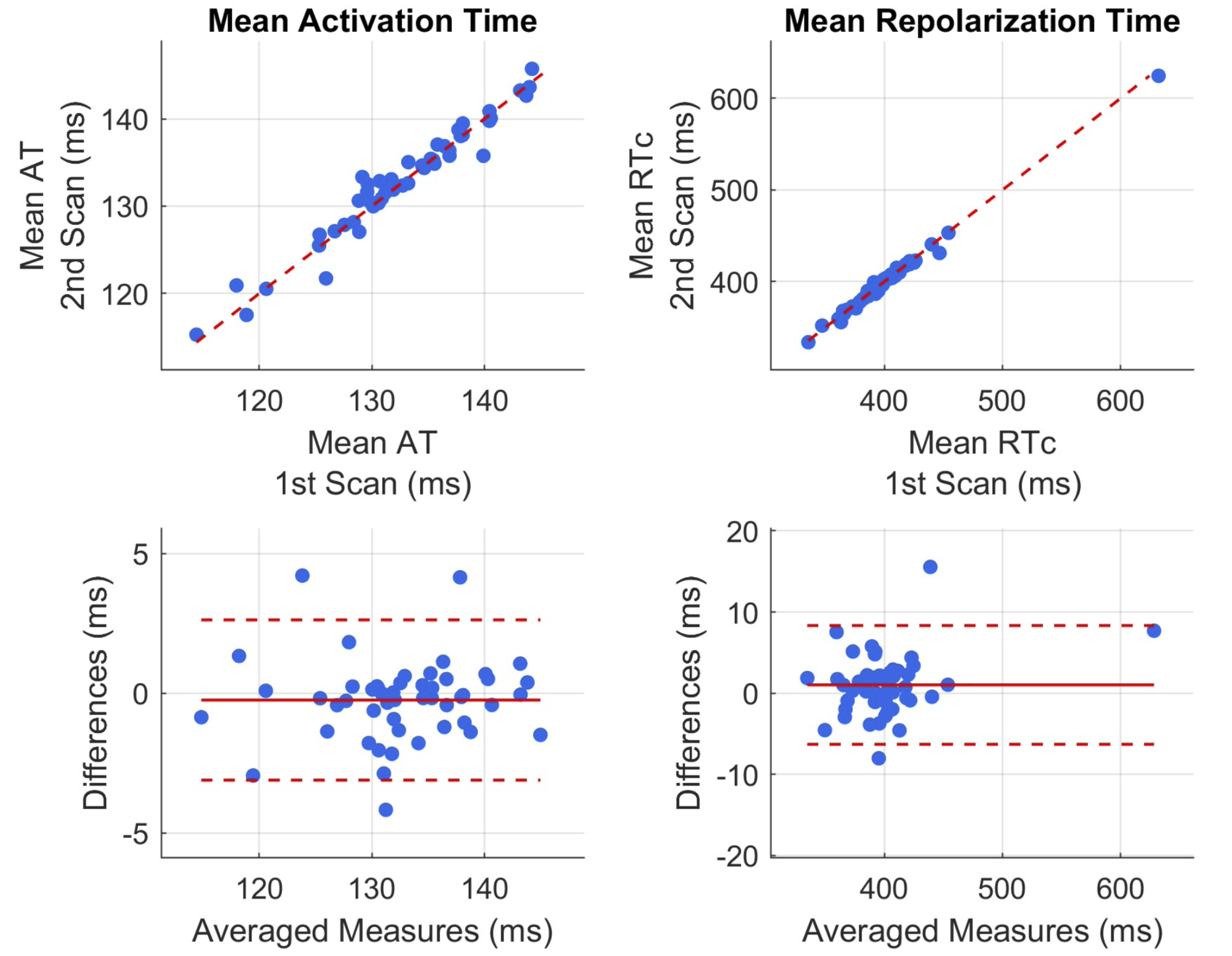

Supplement: Supplementary file 2 — Additional file 2: Figure S2. Intraclass correlation coefficient and Bland–Altman plots for automatic vs manually generated mirror vests for landmark localisation. Top panels show the intra-class correlation coefficient plots while bottom panels show the Bland–Altman plots comparing global mean AT (ICC 0.95 [0.92–0.97] and RT (ICC 0.92 [0.87–0.95]) across automatically generated mirror vest for landmark localisation vs manual imputation (n = 50). Abbreviations as in previous Figs. 1 and 7. [file 12968_2023_980_MOESM2_ESM.png]
