# Supplementary material for: Technical development and feasibility of a reusable vest to integrate cardiovascular magnetic resonance with electrocardiographic imaging
Source: J Cardiovasc Magn Reson. 2023 Dec 4;25:73. doi: 10.1186/s12968-023-00980-7 (PMC10694972; doi:10.1186/s12968-023-00980-7)
Supplement: Supplementary file 3 — Additional file 3: Table S1. Validation of ‘virtual’ automatically generated mirror vest for landmark localisation vs manual imputation (n = 50) [file 12968_2023_980_MOESM3_ESM.docx]

**Additional file 3: Table S1.** Validation of ‘virtual’ automatically generated mirror vest for landmark localisation vs manual imputation (n=50)

| **Validation parameter** | **Automated v Manual** |
| --- | --- |
| *r*_s_UEG | 0.99 (0.98 – 1.00) |
| *r*_s_AT | 0.92 (0.86 – 0.96) |
| *r*_s_RT | 0.86 (0.82 – 0.91) |
| *r*_s_ARI | 0.84 (0.79 – 0.90) |
| iccUEG | 0.99 (0.97 – 1.00) |
| iccAT | 0.95 (0.92 – 0.97) |
| iccRT | 0.92 (0.87 – 0.95) |
| iccARI | 0.92 (0.86 – 0.95) |
| ∆AT, ms | 0.83 (0.42– 2.08) |
| ∆RT, ms | 1.25 (0.42 – 3.33) |
| ∆ARI, ms | 1.25 (0.42 – 3.75) |
|  |  |

All *p* values (not shown) are statistically significant.

Data are presented as median (IQR).

icc = inter-class correlation coefficient 2-way random effects without interactions.

*r_s_UEG = average spearman’s correlation coefficient across each individual cardiac site (n = 12’937); r_s_AT, r_s_RT and r_s_ARI = average spearman’s correlation coefficient measuring the similarity of the activation, repolarization and ARI sequence per participant (n = 20 in each group); iccUEG = average icc across each cardiac site (n = 12’937); iccAT, iccRT and iccARI = average icc measuring the similarity of the activation, repolarization and ARI sequence per participant (n=20 in each group); ∆AT, ∆RT and ∆ARI = absolute difference in AT, RT and ARI across each individual cardiac site (n=12’937) as presented in ms; UEG, unipolar electrogram; Other abbreviations as in* ***Table 1****.*
